# Efficacy of *Lippia sidoides* Containing‐Dentifrice in Children With Dental Caries: A Clinical Study

**DOI:** 10.1155/ijod/4772820

**Published:** 2026-05-12

**Authors:** Patrícia Leal Dantas Lobo, Cristiane Sá Roriz Fonteles, Lídia Audrey Rocha Valadas, Said Gonçalves da Cruz Fonseca, Francisco Vagnaldo Fechine, Elaine Santos, Maria Elisabete Amaral de Moraes

**Affiliations:** ^1^ School of Pharmacy, Dentistry and Nursing, Federal University of Ceara, Fortaleza, Ceara, Brazil, ufc.br; ^2^ Pediatric Dentistry, UTHealth Houston School of Dentistry, Houston, Texas, USA; ^3^ Department of Preventive and Community Dentistry, School of Dentistry, University of Buenos Aires, Buenos Aires, Argentina, uba.ar; ^4^ Center of Drug Development, School of Medicine, Federal University of Ceara, Fortaleza, Ceara, Brazil, ufc.br

**Keywords:** caries, children, fluoride, *Lippia sidoides*

## Abstract

Dental caries is the most prevalent dental disease, especially in childhood, involved in a dynamic pathological process, rendering a microbial shift towards survival and overgrowth of acidogenic and aciduric bacterial species, in which the interaction of biofilm and exposure to carbohydrates is directly associated with lesion formation. The objective of this study was to compare the efficacy of a dentifrice containing 0.8% *Lippia sidoides* Cham oil (LSO) against *Streptococcus mutans* (*S. mutans*) in children. This was a randomized and controlled study. Forty participants, aged between 6 and 12 years, of both sexes, with the presence of at least one carious lesion, were recruited to participate in the study. Participants were randomly assigned into two different groups. The first group (G1) received topical treatment with 0.8% LSO dentifrice, and the control received fluoridated common dentifrice (G2), for brushing their teeth for 1 min thrice a day. Saliva was collected at seven different times: the baseline (D0), after 5 days (D5), 15 days (D15), 30 days (D30), 60 days (D60), 180 days (D180), and after 1 year/365 days (D365). The LSO dentifrice group showed a significant reduction in *S. mutans* levels at day 30 compared with baseline (*p*  < 0.05), and this reduction was maintained at days 60, 180, and 365. In contrast, the control group demonstrated a temporary reduction at day 30, with bacterial levels returning to baseline by day 365. Intergroup comparisons revealed significantly lower *S. mutans* counts in the LSO group from day 30 onward, with differences persisting through 1 year (*p*  < 0.05). The LSO dentifrice 0.8%, when used thrice daily for 30 days, showed a greater reduction in *S. mutans* for up to 360 days when compared to common fluoride dentifrice in children with caries.

## 1. Introduction

Dental caries is the most prevalent dental disease, involved in a dynamic pathological process, rendering a microbial shift towards survival and overgrowth of acidogenic and aciduric bacterial species, in which the interaction of biofilm and exposure to carbohydrates is directly associated with lesion formation [1]. Bacterial fermentation of these carbohydrates results in acid release [2], and during childhood this process may inflict an extremely negative outcome, with a variety of consequences to a child’s well‐being and esthetics [3].

Clinical studies that investigate the microbial composition of caries lesions commonly reveal a greater prevalence of *Streptococcus mutans* (*S. mutans*) colonies. These bacteria have a great capacity to form biofilm on a solid surface, such as the dental surface, by synthetizing high‐affinity adhesins that target extracellular matrix components. For instance, quantification of *S. mutans* in saliva is still considered an indicator of caries risk, since there is a strong association between the biofilm development and the presence of these bacteria [2]. Thus, treatment and control of caries require a balance between pathological and protective factors in order to prevent or arrest carious lesions. The use of fluoridated dentifrice is considered the main strategy for control and reduce dental caries within the last few decades [4].

Previous studies have documented the activity of formulations containing natural products against the dental biofilm, particularly the cariogenic biofilm [5–8]. *Lippia sidoides* Cham. has a wide antibacterial and antifungal spectrum and is considered safe [9]. *Lippia sidoides* Cham. is a bush with odoriferous leaves, native to northeast Brazil and northern parts of the state of Minas Gerais. The essential oil of this plant contains mainly constituents such as thymol, carvacrol, and other substances, such as phellandrenes, caryophyllene, *p*‐cymene, and myrcene [10]. The use of this essential oil against *S. mutans* has been previously demonstrated by our group [11, 12].

Current evidence demonstrates that patients with active carious lesions may benefit from antimicrobial measures, in addition to the mechanical removal of dental biofilm [13, 14]. Hence, the present study aimed to compare the efficacy of a fluoridated (1000 ppm F^−^) dentifrice containing *Lippia sidoides* Cham. at a previously established concentration of 0.8% [11, 12], to a regular fluoridated dentifrice (1000 ppm F^−^), without this essential oil, against *S. mutans* in the saliva of children with dental caries.

## 2. Materials and Methods

### 2.1. Oil Extraction and Chemical Analysis

Samples of *Lippia sidoides* Cham. were collected at the Natural Products Laboratory at the Federal University of Ceará (UFC, Fortaleza, Brazil) and taxonomically authenticated at the Department of Biology in the same university. The essential oil was extracted via hydrodistillation using a Clevenger‐type apparatus and preserved in glass vials under refrigeration until further utilization. The chemical constituents were identified using a gas chromatographer coupled to a mass spectrometer system (GC–MS, Shimadzu, model QP 5050, Japan).

The main components of the *Lippia sidoides* Cham. oil (LSO) used in this clinical trial were cycloheptatriene (0.98%), benzene (2.07%), caryophyllene (3.59%), and thymol/carvacrol (93.36%). Then the dentifrice was formulated with a concentration of 0.8% LSO (concentration defined by previous steps) [11, 12], and 1000 ppm of *F* ^−^ (sodium fluoride, NaF), rendering 0.74% of the Thy/Car mixture. The dentifrices were produced at the Pharmacotechnical Laboratory of the School of Pharmacy in the same university, being standardized with the same color, odor, and taste (Table 1). Concentration of LSO was defined by previous studies [11, 12].

**Table 1:** Constituents used in the different dentifrices.

LSO: 0.8% LSO dentifrice (1000 ppm F^−^)	Control: Fluoridated regular dentifrice (1000 ppm F^−^)
Carboxymethylcellulose sodium (0.5 g) Sorbitol 70% Silicium dioxide (20 g) Sodium saccharine (0.2 g) Sodium lauril sulfate (0.9 g) Sodium fluoride (0.22 g) Macrogol 400 (3.5 g) *Mentha Arvensis essence* (0.5 g) Polysorbate 80 (7.2 g) Purified water (18.2 g) *Lippia sidoides* essential oil (0.8 g)	Carboxymethylcellulose sodium (0.5 g) Sorbitol 70% Silicium dioxide (20 g) Sodium saccharine (0.2 g) Sodium lauril sulfate (0.9 g) Sodium fluoride (0.22 g) Macrogol 400 (3.5 g) *Mentha Arvensis essence* (0.5 g) Polysorbate 80 (7.2 g) Purified water (18.2 g)

### 2.2. Study Design and Patient Recruitment

This is a randomized, controlled, and double‐blind clinical study that followed consort checklist.

**Figure 1:**
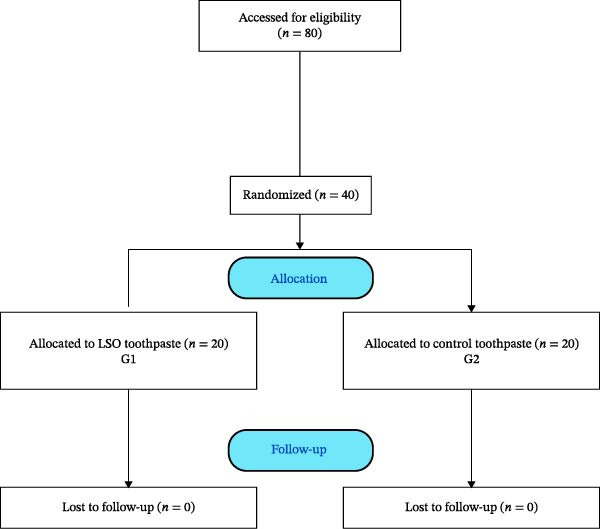
Flow diagram of participants in the clinical trial.

The study protocol was approved by the Ethics Committee of the Federal University of Ceará, Brazil, under number 182/07. Parents or legal guardians of public schools for children were invited to participate in the study and wrote and signed the informed consent. A total of 40 participants were designed to demonstrate the statistical superiority of the dentifrice containing LSO compared with the common dentifrice, considering a power of 90% (*β* = 0.10) and a significance level of 5% (*α* = 0.05). Then 40 children, between 6 and 12 years of age, of both sexes, with at least one carious cavitated or noncavitated lesion, were recruited to participate in the study (Figure 1). As exclusion criteria, patients with a history of allergic diseases, oral mucosal lesions, and those who received antibiotics during the trial were excluded.

### 2.3. Treatment Application

Participants were randomly assigned into two different groups. The first group (G1) received topical treatment with 0.8% LSO dentifrice and the control received fluoridated common dentifrice (G2).

Participants were randomly assigned, by an excel program, to one of two groups (20 children/group). The first group (G1) received topical treatment with 0.8% LSO dentifrice, and the control group (G2) received fluoridated dentifrice without LSO (1000 ppm fluoride). The identification of LSO and control dentifrices was only carried out when the clinical study was concluded.

Before the start of treatment, a clinical examination was performed by one calibrated examiner, using a visual/tactile method to determine the presence of dental caries and the type of lesion that was detected (cavitated or noncavitated). Patients with caries were scheduled for treatment at the Pediatric Dental Clinic of the same university. All patients received the same type of toothbrush and recommendations for oral hygiene and diet to be followed throughout the study. The volunteers brushed their teeth for 1 min three times a day for 30 days.

### 2.4. Saliva Collection and Microbiological Analysis

Saliva was collected at seven different times: the baseline (D0), after 5 days (D5), 15 days (D15), 30 days (D30), 60 days (D60), 180 days (D180), and after 1 year/365 days (D365). Before the collection each patient chewed a piece of 3 × 3‐cm plastic film (Parafilm) for 60 s to stimulate the production of saliva and release the bacteria from the dental biofilm. Samples were collected under the same conditions, operated between 9:00 and 11:00 a.m to minimize the influence of the circadian cycle. Saliva was stored in sterile microcentrifuge tubes (Eppendorf), and samples were prepared for microbiological analysis no longer than 2 h after collection.

For microbiological analysis, a total of 0.1 mL of each sample was added to 0.9 mL of saline solution. All analyses were performed in duplicate, considering two different dilutions (1:10 and 1:100). Thus, the corresponding volume of 10 μL of each dilution was plated onto *Mitis Salivarius-Bacitracin* (MSB) agar medium in triplicates. After incubation at 37°C for 48 h, in jars under microaerophilic conditions, the colonies with morphological characteristics of *S. mutans* were counted, isolated, and biochemically confirmed to be *S. mutans* utilizing mannitol, sorbitol, lactose, raffinose, melibiose, and esculin. After, the bacterial counts were expressed as colony‐forming units per milliliter of saliva (CFU/mL).

Concentrations of *S. mutans* were measured on day 1, prior to use of the dentifrice (defined as the baseline value), and 5, 15, 30, 60, 180, and 365 days after treatment initiation.

### 2.5. Statistical Analysis

For statistical analysis, the Kolmogorov–Smirnov test was used to confirm data normality. Once the normality was confirmed, descriptive statistics, mean, and standard deviation were calculated, and parametric tests were applied. Comparisons between the two treatment groups were made using the *t*‐test for unpaired variables. Analysis of variance for repeated measures, combined with Tukey’s multiple comparison test, was used to compare the different time points within the same group. In all cases, the *α* probability of type I error (significance level) was set at 0.05 (5%), with a two‐tailed *p*‐value  < 0.05 being considered statistically significant. GraphPad Prism statistical software, version 5.00 for Windows (GraphPad Software, San Diego, California, USA, 2007), was used for data analysis and graphing.

## 3. Results

### 3.1. Comparisons Within Groups

**Figure 2:**
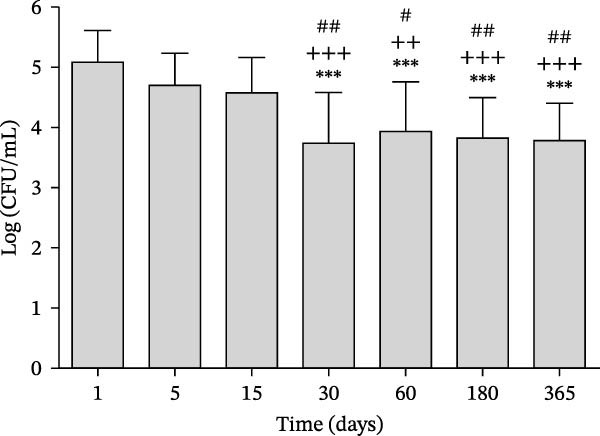
Amount of *S. mutans* expressed as log (number of CFUs per ml of saliva), measured on day 1 (defined as baseline) and days 5, 15, 30, 60, 180, and 365 in the patients in the group treated with LSO toothpaste (control).  ^∗∗∗^ *p* < 0.001 compared to day 1, ^++^ *p* < 0.01 compared to day 5, ^+++^ *p* < 0.001 compared to day 5, ^#^ *p* < 0.05 compared to day 15, ^##^ *p* < 0.01 compared to day 15.

**Figure 3:**
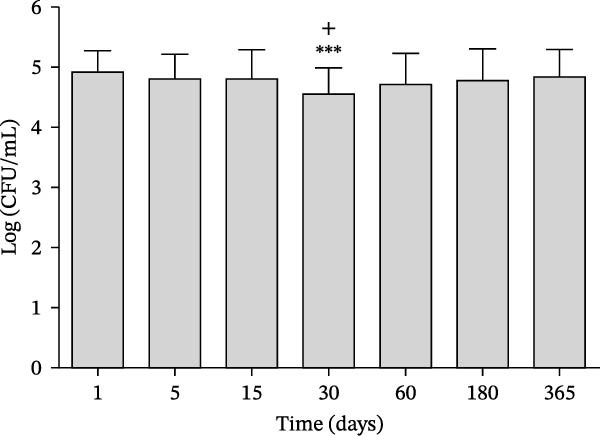
Amount of *S. mutans* expressed as log (number of CFUs per ml of saliva), measured on day 1 (defined as baseline) and days 5, 15, 30, 60, 180, and 365 in the patients in the group treated with LSO toothpaste who completed all the evaluations. ^+^ *p* < 0.05 compared to day 1,  ^∗∗∗^ *p* < 0.001 compared to day 5 and 15.

Figures 2 and 3 illustrate progression over time of the number of colony forming units (CFU) of *S. mutans* in groups treated with regular (control) and LSO dentifrices, respectively. In the LSO group (Figure 2), a significant reduction in the amount of *S. mutans* was observed on day 30 compared with days 1, 5, and 15, but the reduction was maintained on days 60, 180, and 365. In the control group (Figure 3), a significant reduction of *S. mutans* was noted on day 30 compared with days 1, 5, and 15, followed by an increase over the subsequent days, reaching baseline values by day 365.

### 3.2. Comparisons Between Groups

Table 2 shows comparisons of the concentrations of *S. mutans* between the control and the LSO groups, measured on day1 (defined as the baseline value) and days 5, 15, 30, 60, 180, and 365 after treatment. There was a significant reduction in the amount of *S. mutans* in the LSO group compared with the control after day 30, which intensified in the evaluations on days 60, 180, and 365.

**Table 2:** Amount of *S. mutans* expressed as log (number of CFUs per mL of saliva), measured on day 1 (defined as baseline) and days 5, 15, 30, 60, 180, and 365 in the groups treated with both plain dentifrice (control) and LSO dentifrice.

Day	Control		LSO		Significance (*t*‐test)
	Mean (*n*)	Standard deviation	Mean (*n*)	Standard deviation	
1	5.03 (21)	0.40	5.01 (22)	0.70	*p* = 0.9450
5	4.86 (20)	0.37	4.61 (19)	0.52	*p* = 0.0919
15	4.86 (17)	0.41	4.57 (15)	0.55	*p* = 0.1002
30	4.50 (17)	0.71	3.71 (16)	0.77	*p* = 0.0042
60	4.71 (17)	0.43	3.87 (18)	0.69	*p* = 0.0001
180	4.77 (13)	0.51	3.82 (13)	0.59	*p* = 0.0002
365	4.81 (14)	0.43	3.73 (14)	0.57	*p* < 0.0001

*Note:* At each interval, comparisons between the control and the LSO groups were conducted using the unpaired *t*‐test.

The number of patients who showed a confirmed decrease of *S. mutans* in the LSO group was significantly higher than the number of patients who demonstrated similar results in the control (Table 3) on days 30 ( ^∗∗^ *p* = 0.0024), 60 ( ^∗∗^ *p* = 0.0076), 180 ( ^∗^ *p* = 0.0391), and 365 ( ^∗^ *p* = 0.0159).

**Table 3:** Efficacy of the LSO dentifrice vs. plain dentifrice (control) in decreasing the amount of *S. mutans* in saliva, evaluated on days 5, 15, 30, 60, 180, and 365 of the study.

Days	Control *C*/*G* (%)^a^	LSO *C*/*G* (%)	Significance (Fisher’s exact test)
5	0/20 (0.00)	1/19 (5.26)	*p* = 0.4872
15	0/17 (0.00)	1/15 (6.67)	*p* = 0.4688
30	1/17 (5.88)	9/16 (56.25)	*p* = 0.0024
60	0/17 (0.00)	7/18 (38.89)	*p* = 0.0076
180	0/13 (0.00)	5/13 (38.46)	*p* = 0.0391
365	0/14 (0.00)	6/14 (42.86)	*p* = 0.0159

*Note:* The data was analyzed using Fisher’s exact test.

^a^ *C*, Number of patients with clinical improvement (≥25% RrUFC); *G*, total number of patients in the group.

## 4. Discussion

Phytochemicals stand as promising sources in the discovery of novel treatments, with the potential to benefit the pharmaceutical and cosmetic industries. LSO formulations being developed and studied in Dentistry [15–19].

Dentifrices containing essential oils may present the double advantage of providing the resources to both prevent and treat dental caries in high‐caries‐risk individuals [5]. However, it must be considered that daily fluoride dentifrice remains the gold standard in caries control [4, 20]. The presently tested dentifrice formulation contained fluoride in association with LSO and allowed the addition of antibacterial action in the presence of fluoride, which may increase the potential anticaries effect of the formulation.

A number of previous studies have associated fluoridated dentifrice with essential oils [8, 21, 22]. Cagetti et al. [21] tested an experimental dentifrice containing, among other ingredients, fluoride and a mixture of essential oils (*Thymus vulgaris* oil, *Malaleuca alternifolia* oil, *Commiphora myrrha* oil) and compared the efficacy of this experimental formulation with a fluoridated dentifrice, void of antibacterial substances, using the amount of supragingival plaque as their main outcome measure. Their results showed significantly greater antiplaque efficacy of the dentifrice containing the essential essential oil mixture but failed to report on the main components of the oils. In 2019, Valones et al. [22] published a clinical trial comparing the efficacy of an experimental dentifrice containing 5% of a *Rosmarinus officinalis* Linn. extract, without fluoride. The efficacy of this dentifrice was tested against commercially available fluoridated dentifrice and found no significant reduction in plaque index and gingival bleeding after a period of 30 days. Dentifrice containing Brazilian red propolis in addition to fluoride was also previously tested and compared with commercially available fluoridated dentifrice [8]. The dentifrice containing brazilian red propolis demonstrated superior results in the reduction of *S. mutans* and gram‐negative bacteria. In the present study, there was a significant *S. mutans* reduction.

This study evaluated the efficacy of a new dentifrice for controlling biofilm. A 5‐day administration of LSO dentifrice demonstrated a reduction of salivary *S. mutans* after 30 days of treatment, and this reduction persisted, and *S. mutans* levels did not return to baseline concentrations during subsequent analysis (days 30, 60, 180, and 365). Previously, the effect of LSO gel and mouthwash formulations was tested in 37 healthy 6‐to‐12‐year‐old children [11] in order to establish the best formulation and concentration of LSO for future clinical testing. The study resulted in the identification of LSO 0.6% and 0.8% as the safest and most effective doses for testing. Subsequently, the effect of LSO was evaluated and compared with chlorhexidine [12], and among other formulations, the dentifrice presented the most promising results, justifying our choice of formulation in the present clinical trial.

LSO is derived from a plant classified as safe, which makes its addition in dental formulations interesting, without major effects on taste buds [23]. In addition to efficacy, acceptance is also important, and in the case of essential oils a high concentration can result in greater antimicrobial efficacy; however, they are not applicable in oral care products, thus avoiding possible side effects and reducing costs [14, 24]. In the previous step, it was shown that patients who used the LSO paste with a concentration above 0.8% reported a momentary mouth burning [12]. Natural products have been previously tested for use in dentistry due to the need to identify new antibiofilm agents. Hence, LSO constitutes an essential oil with demonstrated biological activity against bacteria involved in the etiology of both dental caries and periodontal disease, in formulations such as mouthwashes, gels, and dentifrice [9, 11, 12, 25]. In the present study, a 30‐day use of regular dentifrice demonstrated a 5.88% reduction of *S. mutans* in saliva versus an almost 10‐fold reduction in the LSO group (56.25%, *p* = 0.0024). Interestingly, this significantly persistent *S. mutans* reduction continued to be observed until D365 in the LSO group, whereas the use of regular dentifrice failed to demonstrate such effect. An earlier study by Albuquerque et al. [9] demonstrated the ability of LSO to hinder *S. mutans* adherence to the wall of the glass tube, in vitro. Thus, the observed long term clinical effects of LSO in the reduction of *S. mutans* in saliva may be the product of its antiadherence activity. In addition, Pereira et al.^25^ [25] evaluated the effect of a gel containing 10% LSO in the control of plaque and gingivitis, comparing a group treated with chlorhexidine and a negative control (placebo).

The authors reported antibiofilm efficacy of the treatment gel following a 3‐month, 3‐times a‐day use of the gel. Another study evaluated the efficacy of using 10% LSO for 3 weeks in the reduction of gingival bleeding. The authors observed a significant difference between baseline results and the last day of treatment [10].

Most current studies on natural antimicrobial substances involve *S. mutans* in their outcomes [13]. Many studies use saliva as a representative medium of the oral ecosystem, since it is present on all surfaces of the oral cavity. Thus, the association of its composition and the presence of caries at different levels is common, since the collection of saliva samples is a simple, effective, and accessible method, justifying the use of this biological material as a biomarker in this study [2, 7].

Several in vitro and some in vivo studies have evaluated the antimicrobial efficacy of LSO on dental biofilm; however, few have investigated its effect in children, the population most affected by dental caries [11, 12]. In addition, most clinical trials in dentistry are related to the effectiveness of restorative materials, with few studies focused on prevention [26]. Regarding formulations with natural products, scientific evidence indicates that clinical trials with caries prevention outcomes are quite heterogeneous, with a low quality in the characterization of the evaluated products and a short follow‐up period, which compromises their reliability and reproducibility [2, 7, 8, 11, 12].

Despite the large number of studies on the biological activity of natural products and molecules in dentistry, few formulations reach the clinical stage, limiting the number of clinical trials and consequently, clinical and scientific evidence [13, 27, 28].

The combination of a fluoridated toothpaste with an essential oil with antimicrobial action may represent a complementary strategy in controlling the disease, especially in children at high risk of caries. Furthermore, since it is a plant considered safe, it may have potential application in public health programs aimed at the child population. However, the study presents important limitations, such as a small sample size and a direct intervention period of only 30 days, despite a 1‐year follow‐up. In addition, the primary outcome was microbiological (reduction of *S. mutans*), without direct evaluation of the incidence or clinical progression of caries lesions.

## 5. Conclusion

The dentifrice LSO 0.8% in this study when used three times daily for 30 days showed a greater reduction in *S. mutans* when compared to regular fluoridated dentifrice for 365 days, which may represent a suitable dosage for children with caries. Further long‐term studies are warranted to establish the long‐term effects of this product.

## Author Contributions

Conceptualization, methodology, investigation, data curation: Patrícia Leal Dantas Lobo. Conceptualization, data curation, writing – review and editing: Cristiane Sá Roriz Fonteles. Data curation, writing original draft – review and editing: Lídia Audrey Rocha Valadas. Methodology, formal analysis: Said Gonçalves da Cruz Fonseca. Supervision, validation, writing – review and editing: Francisco Vagnaldo Fechine. Investigation, data curation: Elaine Santos. Supervision, project administration, funding acquisition, writing – review and editing: Maria Elisabete Amaral de Moraes.

## Funding

This study was supported by the Coordenação de Aperfeiçoamento de Pessoal de Nível Superior.

## Disclosure

Final approval of the version to be published.

## Ethics Statement

This study followed all the ethical rules and Helsinki declaration.

## Conflicts of Interest

The authors declare no conflicts of interest.

## Data Availability

The data that support the findings of this study are available from the corresponding author upon reasonable request.

## References

[1] Sanz M. , Beighton D. , and Curtis M. A. , et al.Role of Microbial Biofilms in the Maintenance of Oral Health and in the Development of Dental Caries and Periodontal Diseases. Consensus Report of Group 1 of the Joint EFP/ORCA Workshop on the Boundaries Between Caries and Periodontal Disease, Journal of Clinical Periodontology. (2017) 44, no. Suppl 18, S5–S11. 28266109 10.1111/jcpe.12682

[2] Valadas L. A. R. , Gurgel M. F. , and Mororó J. M. , et al.Dose-Response Evaluation of a Copaiba-Containing Varnish Against *Streptococcus mutans* In Vivo, Saudi Pharmaceutical Journal. (2019) 27, no. 3, 363–367, 10.1016/j.jsps.2018.12.004, 2-s2.0-85058798953. 30976179 PMC6438705

[3] de Albuquerque L. S. , de Queiroz R. G. , Abanto J. , Strazzeri Bönecker M. J. , Soares Forte F. D. , and Sampaio F. C. , Dental Caries, Tooth Loss and Quality of Life of Individuals Exposed to Social Risk Factors in Northeast Brazil, International Journal of Environmental Research and Public Health. (2023) 20, no. 17, 10.3390/ijerph20176661, 6661. 37681801 PMC10487409

[4] Pitts N. B. , Zero D. T. , and Marsh P. D. , et al.Dental Caries, Nature Reviews Disease Primers. (2017) 3. 10.1038/nrdp.2017.30 28540937

[5] Freires I. A. and Rosalen P. L. , How Natural Product Research Has Contributed to Oral Care Product Development? A Critical View, Pharmaceutical Research. (2016) 33, no. 6, 1311–1317, 10.1007/s11095-016-1905-5, 2-s2.0-84961112556. 26975359

[6] Rodrigues Neto E. M. , Valadas L. A. R. , and Lobo P. L. D. , et al.Dose-Response Evaluation of Propolis Dental Varnish in Children: A Randomized Control Study, Recent Patents on Biotechnology. (2020) 14, no. 1, 41–48, 10.2174/1872208313666190826145453. 31448718

[7] Valadas L. A. R. , Lobo P. L. D. , and Fonseca S. G. D. C. , et al.Dental Varnish in Children: A Clinical Study, Evidence-Based Complementary and Alternative Medicine. (2021) 2021, 6647849. 33833817 10.1155/2021/6647849 PMC8018848

[8] Furtado Júnior J. H. C. , Valadas L. A. R. , and Fonseca S. G. D. C. , et al.Clinical and Microbiological Evaluation of Brazilian Red Propolis Containing-Dentifrice in Orthodontic Patients: A Randomized Clinical Trial, Evidence-Based Complementary and Alternative Medicine. (2020) 2020, no. 1, 10.1155/2020/8532701, 8532701. 32063987 PMC6996680

[9] Albuquerque A. C. L. , Pereira M. S. V. , and Silva D. F. , et al.The Anti-Adherence Effect of Lippia Sidoides Cham: Extract Against Microorganisms of Dental Biofilm, Revista Brasileira de Plantas Medicinais. (2013) 15, no. 1, 41–46, 10.1590/S1516-05722013000100005, 2-s2.0-84876010550.

[10] Rodrigues I. S. , Tavares V. N. , Pereira S. L. , and Costa F. N. , Antiplaque and Antigingivitis Effect of Lippia Sidoides: A Double-Blind Clinical Study in Humans, Journal of Applied Oral Science. (2009) 17, no. 5, 404–407, 10.1590/S1678-77572009000500010, 2-s2.0-70749134877. 19936516 PMC4327664

[11] Lobo P. L. , Fonteles C. S. , and de Carvalho C. B. , et al.Dose-Response Evaluation of a Novel Essential Oil Against Mutans Streptococci In Vivo, Phytomedicine. (2011) 18, no. 7, 551–556, 10.1016/j.phymed.2010.10.018, 2-s2.0-79955690680. 21112195

[12] Lobo P. L. , Fonteles C. S. , and Marques L. A. , et al.The Efficacy of Three Formulations of Lippia Sidoides Cham. Essential Oil in the Reduction of Salivary *Streptococcus mutans* in Children With Caries: A Randomized, Double-Blind, Controlled Study, Phytomedicine. (2014) 21, no. 8-9, 1043–1047, 10.1016/j.phymed.2014.04.021, 2-s2.0-84901984341. 24863037

[13] Freires I. , Denny C. , Benso B. , De Alencar S. , and Rosalen P. , Antibacterial Activity of Essential Oils and Their Isolated Constituents Against Cariogenic Bacteria: A Systematic Review, Molecules. (2015) 20, no. 4, 7329–7358, 10.3390/molecules20047329, 2-s2.0-84928651678. 25911964 PMC6272492

[14] Cieplik F. , Wimmer F. , and Muehler D. , et al.Phenalen-1-One-Mediated Antimicrobial Photodynamic Therapy and Chlorhexidine Applied to a Novel Caries Biofilm Model, Caries Research. (2018) 52, no. 6, 447–453, 10.1159/000487815, 2-s2.0-85045070883. 29617682

[15] Santana Neto M. C. , Costa M. L. V. A. , and Fialho P. H. D. S. , et al.Development of Chlorhexidine Digluconate and Lippia Sidoides Essential Oil Loaded in Microemulsion for Disinfection of Dental Root Canals: Substantivity Profile and Antimicrobial Activity, AAPS PharmSciTech. (2020) 21, no. 8, 10.1208/s12249-020-01842-6, 302. 33146782

[16] Barreto J. O. , do Nascimento F. B. S. A. , and Fonseca S. G. D. C. , et al.Microbiological Evaluation of an Experimental Denture Cleanser Containing Essential Oil of *Lippia sidoides* , Biofouling. (2021) 37, no. 1, 117–130, 10.1080/08927014.2021.1885649. 33593175

[17] de Assis E. L. , Silveira F. D. , da Ponte A. V. A. , and Regis R. R. , A Systematic Review of the Potential Effects of *Lippia sidoides* on Dental Plaque and Periodontal Diseases, Planta Medica. (2022) 88, no. 5, 341–355, 10.1055/a-1554-6947. 34598290

[18] Carvalho-Silva J. M. , Teixeira A. B. V. , Valente M. L. D. C. , Shimano M. V. W. , and Dos Reis A. C. , Antimicrobial Activity of Essential Oils Against Biofilms Formed in Dental Acrylic Resin: A Systematic Review of *In Vitro* Studies, Biofouling. (2024) 40, no. 2, 114–129, 10.1080/08927014.2024.2332709. 38538551

[19] Dantas M. V. O. , da Silva Q. P. , and Júnior A. A. , et al.In Situ Gel Containing *Lippia sidoides* Cham. Essential Oil for Microbial Control in the Oral Cavity, Microorganisms. (2025) 13, no. 11, 10.3390/microorganisms13112585, 2585. 41304270 PMC12654771

[20] Karlinsey R. L. and Pfarrer A. M. , Fluoride Plus Functionalized β-TCP: A Promising Combination for Robust Remineralization, Advances in Dental Research. (2012) 24, no. 2, 48–52. 22899679 10.1177/0022034512449463 PMC3706171

[21] Cagetti M. G. , Strohmenger L. , Basile V. , Abati S. , Mastroberardino S. , and Campus G. , Effect of a Dentifrice Containing Triclosan, Cetylpyridinium Chloride, and Essential Oils on Gingival Status in Schoolchildren: A Randomized Clinical Pilot Study, Quintessence International. (2015) 46, no. 5, 437–445. 25646169 10.3290/j.qi.a33530

[22] Valones M. A. A. , Silva I. C. G. , Gueiros L. A. M. , Leão J. C. , Caldas Jr A. F. , and Carvalho A. A. T. , Clinical Assessment of Rosemary-Based Toothpaste (*Rosmarinus officinalis* Linn.): A Randomized Controlled Double-Blind Study, Brazilian Dental Journal. (2019) 30, no. 2, 146–151, 10.1590/0103-6440201902164, 2-s2.0-85064722833. 30970057

[23] Khan S. T. , Khan M. , and Ahmad J. , et al.Thymol and Carvacrol Induce Autolysis, Stress, Growth Inhibition and Reduce the Biofilm Formation by *Streptococcus mutans* , AMB Express. (2017) 7, no. 1, 10.1186/s13568-017-0344-y, 2-s2.0-85013778825, 49. 28233286 PMC5323333

[24] Silva M. D. A. , Valadas L. A. R. , and Júnior F. J. G. , et al.Perception and Adverse Effects of Patients After Using Propolis-Containing Dentifrice, Journal of Young Pharmacists. (2019) 11, no. 4, 421–423, 10.5530/jyp.2019.11.86.

[25] Pereira S. L. , Praxedes Y. C. , Bastos T. C. , Alencar P. N. , and da Costa F. N. , Clinical Effect of a Gel Containing Lippia Sidoides on Plaque and Gingivitis Control, European Journal of Dentistry. (2013) 7, no. 1, 28–34. 23408652 PMC3571506

[26] Levey C. , Innes N. , Schwendicke F. , Lamont T. , and Göstemeyer G. , Outcomes in Randomised Controlled Trials in Prevention and Management of Carious Lesions: A Systematic Review, Trials. (2017) 18, no. 1, 10.1186/s13063-017-2256-1, 2-s2.0-85032742082, 515. 29096680 PMC5669005

[27] Gbinigie O. , Onakpoya I. , Spencer E. , McCall MacBain M. , and Heneghan C. , Effect of Oil Pulling in Promoting Oro Dental Hygiene: A Systematic Review of Randomized Clinical Trials, Complementary Therapies in Medicine. (2016) 26, 47–54, 10.1016/j.ctim.2016.02.011, 2-s2.0-84959440554. 27261981

[28] Chinsembu K. C. , Plants and Other Natural Products Used in the Management of Oral Infections and Improvement of Oral Health, Acta Tropica. (2016) 154, 6–18, 10.1016/j.actatropica.2015.10.019, 2-s2.0-84946434290. 26522671

